# TriSpectraKAN: a novel approach for COPD detection via lung sound analysis

**DOI:** 10.1038/s41598-024-82781-1

**Published:** 2025-02-21

**Authors:** Abhinav Roy, Bhavesh Gyanchandani, Aditya Oza, Anurag Singh

**Affiliations:** IIIT Naya Raipur, Naya Raipur, India

**Keywords:** TriSpectraKAN, Mel-frequency cepstral coefficients (MFCC), Mel-spectrograms (MSpec), Chromagram (Croma), Diagnosis, Respiratory tract diseases, Machine learning, Medical research

## Abstract

This study aims to create an automated, accessible, and cost-effective diagnostic tool for chronic obstructive pulmonary disease (COPD). Traditional diagnostic methods are expensive, time-consuming, and require specialized equipment. The proposed TriSpectraKAN model leverages audio-based lung sound features to improve early diagnosis. TriSpectraKAN is a hybrid model combining spectral features and the Kolmogorov–Arnold Network (KAN) to analyze lung sounds using Mel-frequency cepstral coefficients (MFCCs), chromagram, and Mel spectrograms. Each sub-model focuses on a different audio feature, capturing unique sonic signatures. These features are merged through a hybrid network for comprehensive analysis. The model, trained on a COPD dataset, was deployed on a Raspberry Pi for real-time use. TriSpectraKAN achieved 93% accuracy, an F1 score of 0.98, precision of 0.97, and recall of 0.98. This multimodal approach captured a broad range of lung sound features, improving diagnosis accuracy compared to traditional methods. The integration of multiple audio features in TriSpectraKAN enhances COPD diagnosis, demonstrating the potential of AI and machine learning to transform respiratory disease diagnosis through accessible tools.

## Introduction

Chronic obstructive pulmonary disease (COPD) represents a significant global health challenge, casting a long shadow over populations worldwide. Characterized by progressive airflow limitation in the lungs, it not only compromises the quality of life for millions but also carries a substantial risk of premature death. While tobacco smoking reigns as the predominant culprit, accounting for up to 90% of cases in many regions, it’s crucial to acknowledge the complex interplay of environmental, occupational, and genetic factors that fuel this multifaceted disease. Understanding the broader tapestry of risk factors beyond just smoking, holds the key to effective prevention, early diagnosis, and improved disease management for individuals across the globe^1^. Early diagnosis is crucial for improving the lives of those affected by COPD. However, current diagnostic methods often require specialized equipment and trained personnel, posing accessibility and cost challenges in primary care settings. This highlights the urgent need for a reliable, accurate, and automated COPD detection method that can readily integrate into primary care, paving the way for earlier intervention and improved patient outcomes.

The recent surge in interest in machine learning for automated medical diagnosis extends to chronic obstructive pulmonary disease (COPD) detection. While convolutional neural networks (CNNs) have excelled in analyzing medical images, audio analysis presents a promising alternative. Breathing and cough sounds harbor valuable respiratory health information, as demonstrated by studies utilizing CNNs^2^. For example, a 2020 study achieved a 98.92% weighted accuracy in classifying respiratory diseases from lung sounds using a lightweight CNN. This study was focussed on binary classification with their own dataset. Another work^3^ reported in 2022 proposed a Hybrid CNN-LSTM Network and employed a Focal Loss Function that attained a good accuracy in determining illness severity. Further, a study reported in Ref.^4^ compared Deep CNN to instance learning for copd identification using ct images.

Despite these encouraging findings using lung sound samples, most existing research on COPD diagnosis and prediction relies solely on imaging data, which is relatively computationally extensive as compared to lung sound sample analysis.To address this gap and propose a potential alternative, we introduce a novel mixed CNN model that fuses features from both audio and clinical data for improved prediction accuracy and accessibility. Additionally, the model has been deployed on a Raspberry Pi, demonstrating its potential for low-cost, portable, and real-time COPD diagnosis.

By evaluating our model’s performance against traditional machine learning algorithms on a dataset given by expert clinicians of COPD patients and healthy controls, we aim to:

- Demonstrate the effectiveness of using specific audio signals spatial representation for classification data for COPD prediction.
- Using Kolmogorov–Arnold Networks, we replaced traditional multi-layer perception, achieving superior accuracy and generalizability of our developed proposed model called TriSpectralKAN compared to the existing methods.
- The study leverages lung sound recordings from healthy and COPD subjects across three publicly available databases. This combined dataset is utilized to develop a classification model that can identify COPD-affected cases based solely on their lung sounds. Furthermore, the model was successfully deployed on a Raspberry Pi, highlighting its capability for real-time, portable diagnostics in resource-constrained environments.

The rest of the paper is organized as follows.

“Dataset description” section describes and visualize the employed dataset in detail, “Methodology” section elaborates the proposed methodology including preprocessing steps and detailed technical modules, proposed architecture followed by performance metrics description. Qualitative and quantitative results are discussed “Data preprocessing” section followed by conclusion in “Conclusion” section.

## Related works

Several studies have delved into leveraging machine learning algorithms to diagnose and predict COPD. Several studies have tried the use of machine learning (ML) algorithms for diagnosing and predicting chronic obstructive pulmonary disease (COPD). Wang et al. employed Convolutional Neural Networks (CNNs) to distinguish COPD patients from healthy controls using CT scans, achieving an accuracy of 85.7%^5^. Li et al. examined key variables like age, weight, BMI, and waist circumference, providing insights into their relationship with COPD^6^. COPD symptoms such as wheezing and reversible airway obstruction have been noted, though factors like advanced age and smoking history can complicate diagnosis in asthma patients^7,8^.

Serbes et al. developed an algorithm for classifying respiratory sounds, achieving a 49.86% accuracy, highlighting potential for improvement in noise management and dataset complexity^9^. Jakov et al.’s approach using hidden Markov models and Gaussian mixture models scored 39.56 in the ICHBI Challenge, indicating the need for better noise reduction techniques and model interpretability^10^. Rill et al.’s NMRNN model performed well on a specific dataset but lacked generalizability and comprehensive evaluation metrics^11^.

Chambers et al. explored respiratory sound classification, achieving 85% accuracy with their patient-level assessment model, though lacking precision and recall measures^12^. Liu et al. used CNNs to detect adventitious respiratory sounds, showing significant performance variability across datasets and suggesting potential overfitting^2^.

Deep learning has been extensively applied to respiratory disease classification and diagnosis^13–15^. Ma et al. used the LungBRN architecture but lacked validation in diverse contexts^13^. Acharya et al. proposed a deep CNN-RNN model, achieving moderate accuracy but with high computational complexity^14^. Demir et al. introduced a pretrained CNN for lung sound classification but did not provide detailed performance metrics^15^.

One study achieved 81.62% accuracy on the ICBHI 2017 dataset using a CNN model applied to both public and pediatric auscultation data, showing that combining demographic-specific data can improve diagnostic performance, especially for pediatric patients where sound profiles vary^16^. Another investigation leveraged the ICBHI dataset to test multiple algorithms, including CNNs, SVMs, RNNs, and KNNs, achieving accuracy rates between 80 and 90% and demonstrating that deep learning methods consistently outperform traditional auscultation^17^.

The paper^18^ presents a self-supervised model that incorporates Signal-to-Noise Ratio (SNR) prediction to manage the effects of noisy lung sounds, showing improvements in sound clarity, which are crucial for accurate respiratory assessments. This model aligns with the objectives of the King Abdullah Dataset^19^ by emphasizing the importance of clean sound acquisition and sound phase correction in challenging acoustic conditions. Furthermore, Ref.^20^ introduced the BRACETS dataset, a bimodal database combining respiratory sounds with Electrical Impedance Tomography (EIT) data for differential respiratory diagnosis. This pioneering effort provides a robust multi-modal platform, achieving significant classification accuracy across multiple respiratory disease types and highlighting the potential for multi-modal datasets like King Abdullah’s in advancing respiratory diagnostics.

Recent advancements include the use of audio data for respiratory disease detection. Samiul Based Shuvo et al. used lung auscultation sounds with a lightweight CNN, outperforming larger models like VGG16^21^. Xu et al. achieved high accuracy in COPD detection using deep CNNs on CT images^4^. Subrato Bharati et al. introduced a CNN-VGG-data-augmentation-STN hybrid framework for efficient chest image analysis^22^. Georgios Petmezas et al. implemented a hybrid model combining CNN and LSTM for lung sound classification^3^. While CT-based approaches for chronic bronchitis and emphysema are effective, they face limitations in visualizing and predicting COPD progression due to subjective assessments and expert variability^23,24^. Overall, these methods show promise but require further enhancement for more accurate and advanced diagnostic outcomes, reshaping respiratory disease management in clinical and remote settings.

This study proposes a KAN model designed to blend audio and clinical prediction data for COPD prediction. The model incorporates three distinct data types: MFCC features, chroma features, and Mel-spectrogram features derived from respiratory sound recordings. We evaluate the model’s effectiveness using an openly available dataset containing both COPD patients and healthy individuals. Our results demonstrate that the model outperforms traditional machine learning methods, highlighting its potential as a valuable diagnostic tool for medical practitioners.

## Dataset description

The study evaluated it’s proposed methodology using a combination of the ICBHI 2017 dataset^25^, the King Abdullah University (KAU) dataset, and the Respiratory Database@TR (RD@TR). These are publicly available datasets are widely used in studies focused on classifying respiratory disorders. They include a diverse range of respiratory sounds from individuals with varying health conditions, ages, and clinical states, providing a comprehensive foundation for our research.

Especially, the datasets are carefully preprocessed, with background noise removed and respiratory cycles expertly segmented to isolate pertinent sounds. This meticulous attention to detail expedites research, allowing targeted study of core respiratory sound data and enabling the creation of accurate machine-learning algorithms for the classification of respiratory disorders.

The Table 1 is about the characteristics of the respiratory datasets used.

**Table 1:** Summary of dataset characteristics.

Category	ICBHI 2017 challenge database	Chest wall lung sound database (CWLSD)	Respiratory database@TR (RD@TR)
Dataset origin	Created by research teams in ICBHI 2017 respiratory sound database^25^	Publicly available repository from King Abdullah University Hospital^19^	Publicly available database with recordings from different COPD severity levels^26^
Number of recordings	920 annotated audio recordings, varying lengths (10 s to 90 s)	336 lung sound signals collected from 112 subjects	12-channel recordings from subjects with different COPD severity levels
Patient data	From 126 patients of all age groups	112 subjects with a wide spectrum of respiratory diseases	Subjects with different COPD severity levels, ranging from COPD0 to COPD4
Recording duration	5.5 h of audio recordings	10 to 50 s per audio signal	Minimum 17 seconds of recording
Diagnosis (classes)	URTI, COPD, asthma, LRTI, bronchiectasis, pneumonia, bronchiolitis	Asthma, COPD, bronchiolitis obliterans, heart failure, pulmonary fibrosis, etc.	Different COPD severity levels
Respiratory cycles	6898 cycles, with 1864 with crackles, 886 wheezes, and 506 having both	Not specified	Not specified
Noise simulation	Contains clean and noisy recordings; noisy recordings simulate real-life conditions	Not specified	85% of ambient noise is reduced from the lung sound recordings.
File formats	.wav files	.wav files and annotation .txt files	Not specified
Sampling rate	Not specified	4 kHz using Littmann 3200 digital stethoscope	4 kHz using Littmann 3200 digital stethoscope

### Spatial representation of the dataset

**Fig. 1:**
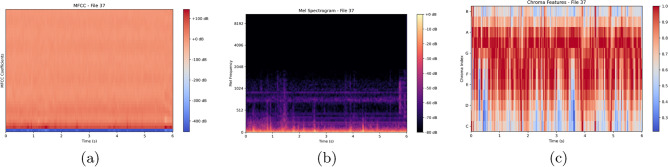
Plots showing the spatial representation of data sample.

The spatial representation of our dataset is crucial for understanding the inherent patterns and characteristics within the audio data. In Fig. 1, we present three distinct representations of the audio using MFCC, melSpectogram, and Chromagram features, respectively. These representations offer visual insights into the frequency and temporal dynamics of the audio signals.

These spatial representations serve as valuable tools for gaining insights into the structure and content of our dataset, aiding in the development and refinement of models for effective analysis and classification of respiratory sounds.

## Methodology

**Fig. 2:**
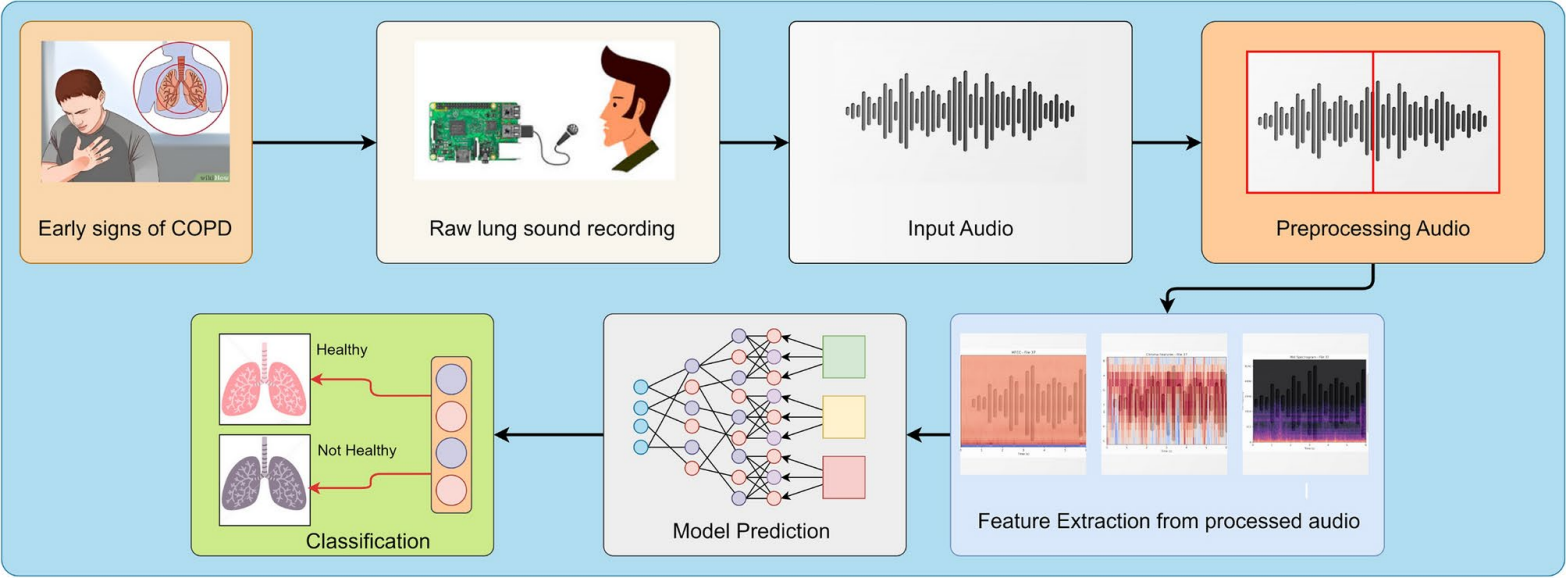
Proposed framework.

The proposed work involves processing three types of features, namely MFCC, chroma, and mel spectrogram, through sub-CNN models. These features are collected to extract accurate non-linear features for precise COPD prediction. The novelty lies in proposing three multi-modal COPD features and a hybrid model that fuses them for reliable and robust predictions. A complete flow diagram of proposed approach is shown in Fig. 2.

In the first stage, feature extraction models were developed for MFCC, chroma, and mSpec inputs, each designed with convolutional and pooling layers. After establishing the feature extraction models, the extracted features from all three models were integrated using the concatenate layer to create a unified representation. This comprehensive representation incorporated relevant information from MFCC, chroma, and mSpec inputs.

To construct the model named “TriSpectralKAN”, input layers representing MFCC, chroma, and mSpec were combined with the output layer of the classification network. This integration facilitated the simultaneous processing of multiple input types for accurate classification predictions.

The hybrid model was compiled with sparse-categorical cross-entropy loss, Nadam optimizer, and accuracy metric. With the learning rate being updated when the training reached on a plateau. The training utilized available data with suitable callbacks for monitoring and early stopping.

Detailed information on model architectures, hyperparameters, and training procedures is available in the respective sections of the research paper.

### Data preprocessing

**Fig. 3:**
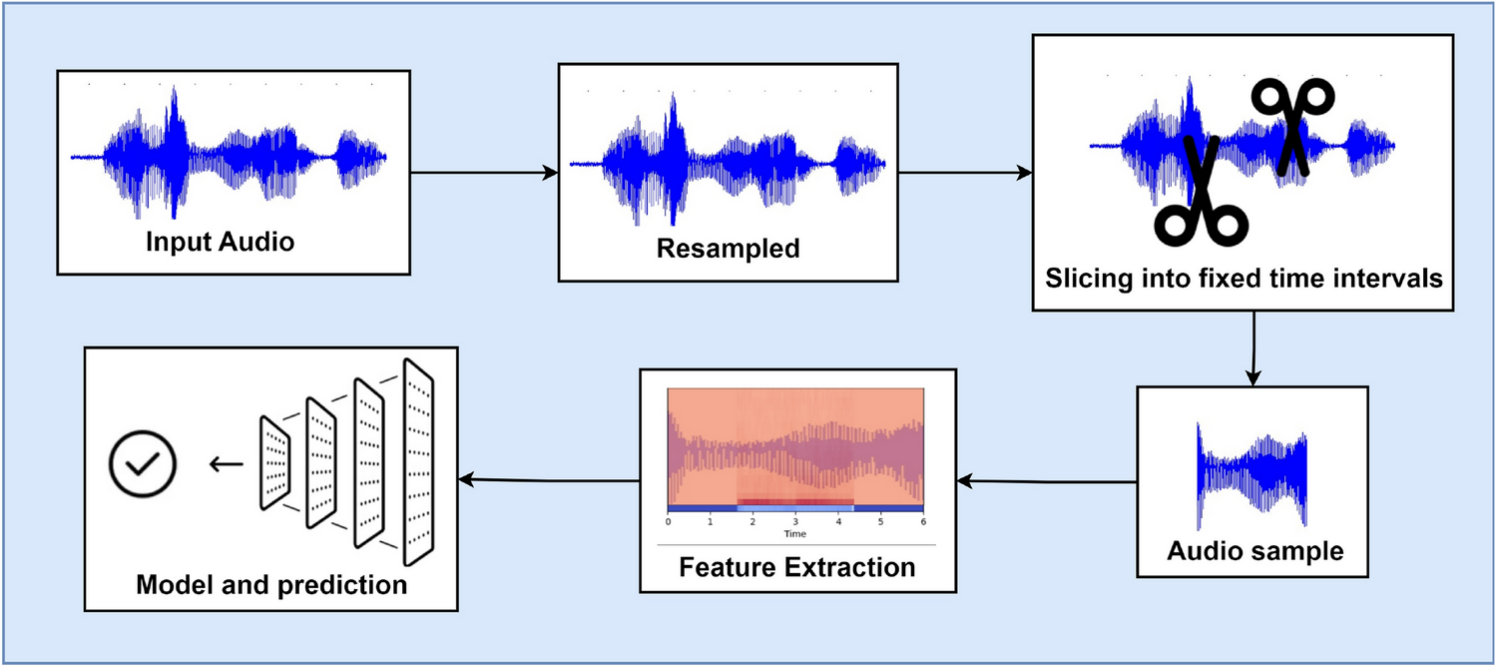
Preprocessing.

Figure 3 demonstrates the process of audio resampling and trimming or padding, followed by feature extraction, enabling the standardization of audio data to a fixed length and the derivation of pertinent features crucial for the subsequent classification of the audio signals. The process begins with taking raw audio samples as input, which are then resampled to a standardized frequency of 22,050 Hz. This resampling step ensures that all audio inputs are aligned to the same sampling rate, facilitating uniform processing across the datasets.

Since we are only interested in sections of the audio that contain respiratory cycles, we extract specific segments based on start and end times noted in our dataset. By isolating these respiratory cycles, we eliminate unnecessary portions of the audio, focusing solely on the relevant sounds for all datasets.

**Fig. 4:**
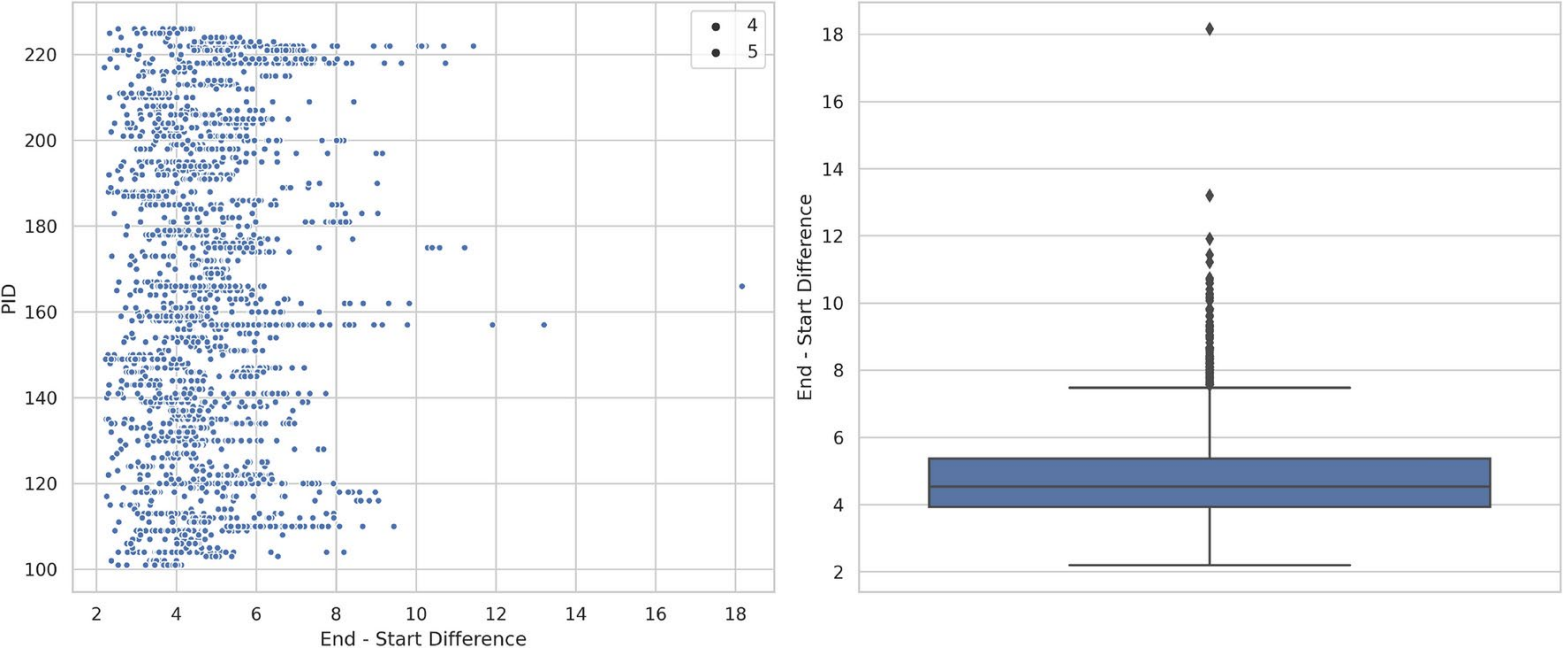
Scatter plot and boxplot showing duration of segments.

Next, each extracted audio segment is adjusted to a fixed length of six seconds, which we determined to be optimal based on our analysis of cycle durations as seen in Fig. 4, the respiratory cycles last for about 6 s in most cases. This fixed-length standardization ensures that the audio input to the model remains consistent in size. For segments shorter than 6 s, we apply zero padding, adding silence to the audio clip to meet the length requirement. Conversely, for segments longer than 6 s, the excess portion is trimmed.

Finally, because some audio files contain multiple respiratory cycles, multiple 6-s segments are saved from a single file if needed. This preprocessing workflow-resampling, segment extraction, length standardization, and padding or trimming-prepares the audio data to be fed into the CNN model, ensuring a uniform input structure that enhances classification accuracy.

### Feature extraction and modelling

The methodology initiates with the extraction of three distinctive acoustic features—Mel-frequency cepstral coefficients (MFCC), Chromagram (chroma), and Mel spectrogram (mSpec)—from the raw audio data. Each of these features undergoes computation using dedicated models: the MFCC model, the Chroma model, and the mSpec model. These models are designed to capture specific aspects of the audio signal and are constructed with a series of convolutional and pooling layers, complemented by batch normalization and activation functions.

The MFCC model employs convolutional layers for Mel-frequency cepstral coefficient pattern analysis, pooling layers for downsampling, and batch normalization for stable training. Activation functions introduce non-linearity for learning complex data relationships. Similarly, the Chroma model focuses on Chroma features, utilizing convolutional layers for spatial analysis, pooling layers for dimensionality reduction, batch normalization for stable activations, and activation functions for capturing intricate patterns. The mSpec model extracts features from the Mel spectrogram using convolutional layers for frequency analysis, pooling layers for effective reduction, batch normalization for stability, and activation functions for understanding nuanced information.

**Fig. 5:**
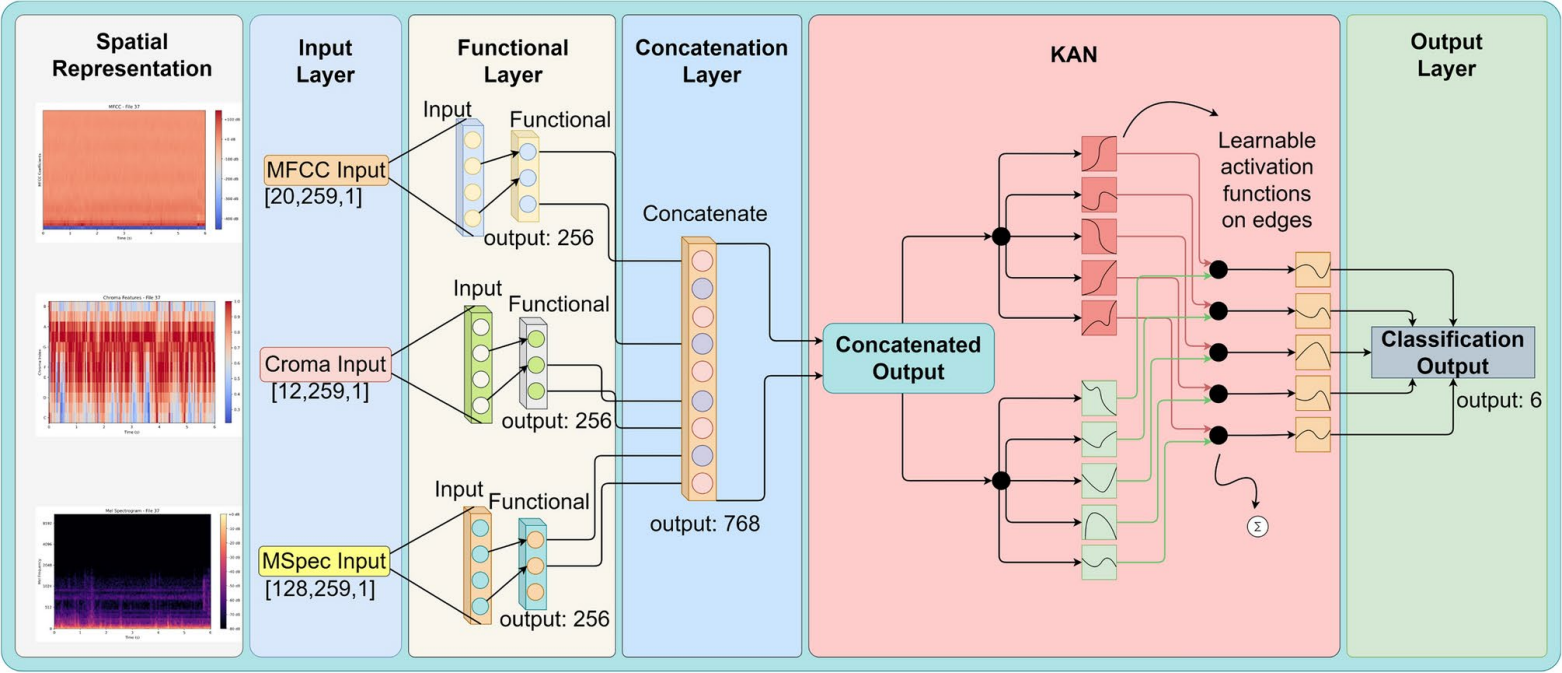
Proposed feature extraction and TriSpectraKAN structure.

A detailed representation of our neural network is shown in Fig. 5. The MFCC head, Chroma head, and MSpec head are the three heads that make up the neural network. These heads match the MFCC, Chroma, and MSpec features that were previously extracted, in that order.

The TriSpectraKAN model combines the MFCC, chroma, and mSpec features through multi-modal fusion, leveraging their distinct characteristics to enhance the classification performance. The fusion of the features allows for capturing different aspects of the audio signals, leading to improved discrimination between audio classes.

### KAN

Kolmogorov–Arnold networks (KANs) are rooted in the Kolmogorov–Arnold representation theorem, which plays a pivotal role in the fields of ergodic theory and dynamic systems. This theorem asserts that any continuous multivariate function $f$ defined over a bounded domain, depending on variables $x = [x_1, x_2, \ldots , x_n]$, can be decomposed into a finite composition of simpler, continuous univariate functions. Specifically, a smooth and continuous function $f(x) : [0,1]^n \rightarrow \mathbb {R}$ can be represented through the superposition of these univariate functions. KANs leverage this theorem by utilizing learnable activation functions instead of fixed ones, setting them apart from traditional neural networks like Multi-Layer Perceptrons (MLPs). Unlike MLPs, where activation functions are fixed and associated with neurons, KANs employ activation functions that are parametrized as splines and associated with the network’s edges (weights). This innovative approach not only allows KANs to efficiently approximate complex functions but also enhances their interpretability and accuracy in tasks involving high-dimensional data. Formally this can be expressed by the finite superposition of univariate functions as follows:

1 $$\begin{aligned} f(x) = \sum _{i=1}^{2n+1} \varPhi _i \left( \sum _{j=1}^{n} \varphi _{i,j}(x_j) \right) , \end{aligned}$$

where $\varPhi _i : \mathbb {R} \rightarrow \mathbb {R}$ and $\varphi _{i,j} : [0,1] \rightarrow \mathbb {R}$ are referred to as the outer and inner functions, respectively. Initially, this theorem appears to be useful for machine learning since it simplifies the job of learning a function of high-dimension to that of a polynomial number of one-dimensional functions. However, these one-dimensional functions may have non-smooth properties, making them challenging to understand in real situations. As a result, this theorem has been typically disregarded in machine learning, regarded as theoretically sound but practicable. Surprisingly, recent theoretical advancements in Ref.^27^ have revived interest in this theorem, leading to new network architectures based on the Kolmogorov–Arnold theorem. The authors of Ref.^27^ found that the equation above has two layers of nonlinearities, including 2n + 1 terms in the middle layer. The aim is to determine the inner univariate functions $\varphi _{i,j}$ and outer functions $\varPhi _i$ that resemble the provided function. B-splines can be used to approximate one-dimensional inner functions. A spline is a smooth curve created by a series of control points or knots. It is used to interpolate or approximate data points smoothly and continuously. Splines are defined by their order k (often k = 3), which refers to the degree of polynomial functions used to interpolate between control points.The number of intervals, represented by G, is the number of segments or subintervals connecting neighboring control points. In spline interpolation, these segments connect the data points to create a smooth curve (with G+1 grid points). Although other forms of splines could potentially be examined, this strategy is given by Ref.^27^.

A two-layer network with activation functions at the edges and nodes carrying out basic summing can be used to illustrate Eq. (1). Smooth splines cannot adequately approximate any arbitrary function with this two-layer network due to its oversimplification. Thus, a generalized architecture with deeper and wider KANs is proposed in Ref.^27^, which expands on these concepts.

A KAN layer is characterized by a matrix $\varPhi$, which consists of univariate functions $\{ \varphi _{i,j}(\cdot ) \}$ with $i = 1, \ldots , N_{\text {in}}$ and $j = 1, \ldots , N_{\text {out}}$. Here, $N_{\text {in}}$ and $N_{\text {out}}$ represent the number of inputs and outputs, respectively, and $\varphi _{i,j}$ are the trainable spline functions as previously described. According to this definition, the Kolmogorov–Arnold representation theorem can be implemented as a two-layer KAN. The inner functions form a KAN layer with $N_{\text {in}} = n$ and $N_{\text {out}} = 2n + 1$, while the outer functions form another KAN layer with $N_{\text {in}} = 2n + 1$ and $N_{\text {out}} = 1$.

The shape of a KAN can be defined as $[n_1, \ldots , n_{L+1}]$, where $L$ is the number of layers in the KAN. The Kolmogorov–Arnold theorem corresponds to a KAN with the shape $[n, 2n+1, 1]$. A deeper, more generic KAN can be expressed as the composition of $L$ layers:

2 $$\begin{aligned} y = \text {KAN}(x) = (\varPhi _L \circ \varPhi _{L-1} \circ \ldots \circ \varPhi _1)x. \end{aligned}$$

All operations in KANs are differentiable, enabling their training via backpropagation. Although KANs are based on an elegant mathematical framework, they are fundamentally combinations of splines and MLPs, leveraging each other’s strengths while mitigating their weaknesses. Splines are proficient at approximating low-dimensional functions and facilitate transitions between different resolutions, yet they struggle with dimensionality issues due to their inability to effectively exploit compositional structures. Conversely, MLPs have fewer dimensionality problems because of their feature-learning capabilities but tend to be less accurate than splines in low-dimensional contexts since they are not as effective at optimizing univariate functions. KANs offer two degrees of freedom: they can acquire features akin to MLPs and optimize these features with the precision of splines. Increasing number of layers $L$ or grid dimension $G$ escalates the number of parameters and, subsequently, the network’s complexity. This methodology provides an alternative to conventional deep learning models that predominantly use MLP architectures, thus motivating the advancement of this research.

### Model specifications and parameters

In this section, we provide a comprehensive overview of the specifications and parameters of the models used in our study. The models are designed to classify audio signals based on their extracted features. We outline the architecture of each model, detailing the layers, their types, and their respective parameters.

Two models are considered in this study. The first model is a Hybrid KAN (TriSpectraKAN) in Table 2 tailored for processing audio features, while the second Hybrid CNN model in Table 3 incorporates CNN for comparison. The following tables summarize the key specifications and parameters for each model, including information about the type of each layer, the number of units, and parameters.

**Table 2:** Hybrid KAN information.

Layer	Output shape	Param #
mfcc input	(20, 259, 1)	0
Chroma input	(12, 259, 1)	0
mspec input	(128, 259, 1)	0
mfccModel	(None, 256)	144,352
cromaModel	(None, 256)	86,528
mSpecModel	(None, 256)	144,352
Concatenate	(None, 768)	0
KAN input	(None, 768)	0
Hidden layer	(None, 769)	785,888
Output layer	(None, 6)	46,140
Total params		1,207,257
Total FLOPs		1.56 M

**Table 3:** Hybrid CNN information.

Layer	Output shape	Param #
mfcc input	(20, 259, 1)	0
Chroma input	(12, 259, 1)	0
mspec input	(128, 259, 1)	0
mfccModel	(None, 256)	144,352
cromaModel	(None, 256)	86,528
mSpecModel	(None, 256)	144,352
concatenate	(None, 768)	0
Dense and dropout	(None, 750)	576,750
Dense and dropout	(None, 250)	187,750
Dense and dropout	(None, 75)	18,225
Dense and dropout	(None, 25)	1900
Fense layer	(None, 6)	156
Total params		1,160,613
Total FLOPs		1.94 M

**Table 4:** Hyperparameters used during training.

Hyperparameter	Value tested	Final value
Spline order	3, 5, 7	3
KAN hidden layer	128, 384, 769	769
Batch size	16, 32, 64	32
Initial learning rate	0.01, 0.001, 0.0001	0.001
Number of epochs	20, 50, 100	50
Optimizer	Nadam, Adam	Nadam

Table 4 summarizes the hyperparameters chosen for training our KAN model. In KAN models, increasing the spline order allows for a more complex model that can better capture intricate patterns in the data. However, this complexity also increases the risk of overfitting. We experimented with spline orders of 3, 5, and 7 and found that while a degree 7 spline quickly led to overfitting, both degree 3 and 5 splines performed similarly, with no significant improvement in accuracy. Therefore, we selected a spline order of 3 as it provides a simpler model with fewer parameters, which reduces computational overhead and minimizes overfitting. The number of parameters in each KAN layer is $O(n_1*n_2*g)$ where g is the spline order and $n_1$ and $n_2$ represent the number of neurons in each respective layer. In contrast the number of parameters in each MLP layer is $O(n_1*n_2)$ where $n_1$ and $n_2$ represent the number of neurons in each respective layer The total FLOPs (Floating Point Operations) in a Hybrid CNN are higher than those in a Hybrid KAN, which can be attributed to KAN’s structure as a parameter-dense model. In KAN, the use of spline basis functions leads to fewer weight connections and, therefore, a reduction in FLOPs. This efficient allocation of parameters to basis functions allows KAN models to use smaller weight matrices, effectively lowering computational demands. As a result, KAN models, like TriSpectraKAN, are well-suited for deployment on edge devices, where computational efficiency is essential.

The learning rate was set to 0.001, with an adaptive reduction that decays the rate upon reaching a performance plateau. Additionally, we implemented early stopping to halt training once learning has effectively concluded, preventing unnecessary computation and further reducing the risk of overfitting. The Nadam optimizer was employed to enhance convergence speed and stability. This combination of hyperparameter choices yielded a balanced model that generalizes well without overfitting.

## Results and discussion

### Evaluation metrics

To evaluate the supremacy of the proposed framework, we have utilized the following statistical performance evaluation metrics^28^ such as accuracy, specificity, sensitivity, precision, and F1-score, which can be obtained from the contingency table or confusion matrix that comprises the ground truth labels and TriSpectraKAN predicted labels . The equations for these metrics are provided as : $accuracy = \frac{TP + TN}{TP + TN + FN + FP},$ $specificity = \frac{TN}{TN + FP},$ $sensitivity= \frac{TP}{TP + FN},$ $precision = \frac{TP}{TP + FP},$ $F1-score = 2 \times \frac{precision \times sensivity}{precision + sensivity},$

where TP, TN, FP, and FN denote the true positive, true negative, false positive, and false negative of classification.

### Evaluation of performance

The combined database contains six classes, with the majority of lung sound samples belonging to the COPD class, making it an imbalanced dataset. We have performed a multi-class classification using the proposed TriSpectraKAN architecture. The six classes considered for classification include Bronchiectasis, Bronchiolitis, COPD, Healthy, Pneumonia, and URTI. The data used for training, validation and testing is taken in the ratio: 70:10:20. TriSpectraKAN model was trained with the following simulation parameters: batch size of 32, learning rate of 0.001, optimizer is nadam and epoch of 50, which were determined using the GridSearchCV-based hyper-parameter optimization technique.

Table 5 shows the mean over the fivefold results. The proposed framework achieves an average accuracy, specificity, sensivity, precision and F1-score of 95.22%, 97.6%, 97.4%, 97.4%, and 97.8%, respectively.

**Table 5:** Evaluation metrics across folds with mean.

Evaluation metrics (%)	Fold number					Mean
	Fold 1	Fold 2	Fold 3	Fold 4	Fold 5	
Accuracy	95.6	94.3	96.0	94.9	95.3	95.22
Specificity	97	98	99	97	97	97.6
Sensitivity	97	97	98	98	97	97.4
Precision	98	97	97	98	97	97.4
F1-score	98	98	98	98	97	97.8

**Fig. 6:**
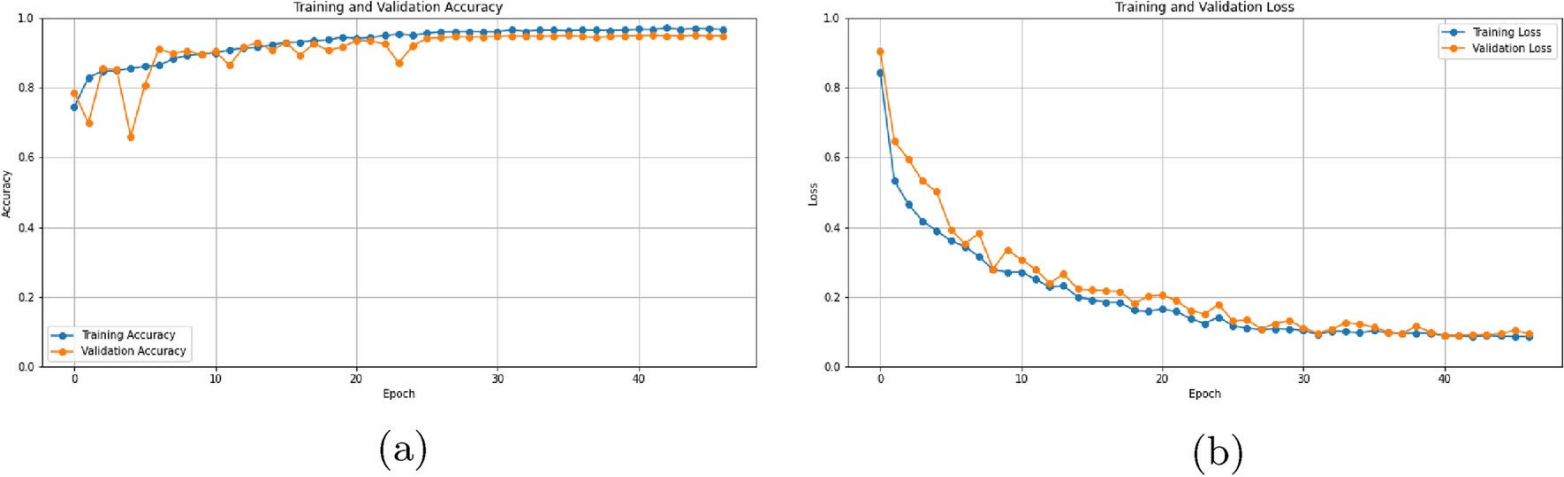
Plots showing the training of model.

Figure 6a, depicts the training and validation accuracy curves of the proposed hybrid CNN model, offering insights into its generalizability. The algorithm was run for 50 epochs. Both curves exhibit gradual slopes, devoid of drastic fluctuations, suggesting good learning from the training data without succumbing to overfitting. Overfitting is given by steep inclines or declines, which signifies the model’s inability to generalize to unseen data. The observed flatness, particularly in the validation accuracy curve, indicates robust generalizability, further supported by the high validation accuracy of around 0.95, indicating more than 95% correct classifications on the validation set. Additionally, the absence of significant fluctuations reinforces the model’s stability and its ability to generate consistent predictions.

Figure 6b delves into the model’s learning dynamics through the visualization of training and validation loss curves. The gentle slopes of both curves, devoid of sharp increases or decreases, imply successful learning without overfitting. The observed gentle slopes suggest a desirable balance between memorizing the training data and learning adaptable patterns, enhancing the model’s ability to generalize to new unseen data. Furthermore, the proximity of the training and validation loss curves throughout the training process bolsters this confidence in generalizability. This close alignment indicates that the model’s performance on unseen data closely resembles its performance on the training data, signifying the transferability of learned patterns to new unseen examples.

In conclusion, both Fig. 6a,b provide compelling evidence that the proposed model achieves a successful balance between learning from the training data and generalizing to unseen data. This characteristic positions the model well for achieving robust and accurate results in real-world applications.

**Fig. 7:**
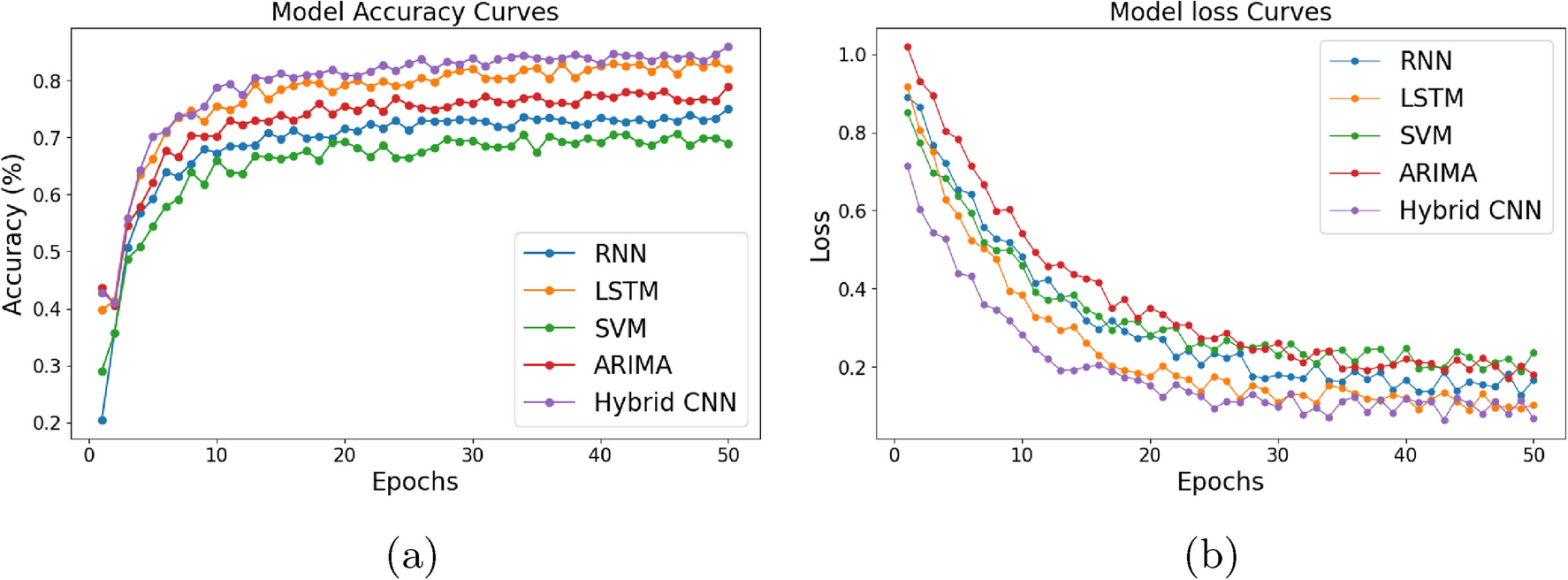
Plots showing the training of model.

While the performance of different models like the RNN, LSTM, SVM, and ARIMA models shows steady improvement as shown in Fig. 7a, the Hybrid CNN performs better than all other models in terms of both accuracy and loss reduction. It starts with a higher accuracy and lower loss, and continues to improve more consistently, showing minimal fluctuations as shown in Fig. 7b.

Despite the strong performance of the Hybrid CNN, the TriSpectraKAN model provides the best results overall. The integration of Kolmogorov–Arnold Networks (KANs) with audio signal inputs (MFCC, MSpec, Chroma) and the advanced architecture of TriSpectraKAN leverages its strengths to achieve superior diagnostic accuracy and robust and consistent performance, surpassing the other models in both accuracy and loss metrics.

After successful traning and validation, the model is tested on unseen data for six class classification. Leveraging the combination of spatial and temporal features, the model exhibited superior capabilities in capturing intricate lung disease patterns within the image data provided through different spatial representations. Through a series of rigorous experiments and evaluations, we observed that our model achieved a maximum accuracy and recall of 93% and 98% for COPD class, respectively followed by Bronchiectasis with the recall of 81%. The comprehensive analysis of the results further revealed the model’s efficacy in handling complex visual data, showcasing its potential for various real-world applications.

The Table 6 presented below provides a detailed breakdown of the precision, recall, and F1 score for our model, offering deeper insights into its performance across different classes and categories.

**Table 6:** Performance metrics for various classes.

Class	Precision	Recall	F-1 score
Bronchiectasis	0.81	0.81	0.81
Bronchiolitis	0.73	0.68	0.70
COPD	0.97	0.98	0.98
Healthy	0.68	0.73	0.7
Pneumonia	0.79	0.79	0.79
URTI	0.70	0.45	0.55

The model performs well on some classes, particularly Chronic Obstructive Pulmonary Disease (COPD), where it achieves an F1 score of 98% , according to Table 6. This means it excels at identifying both healthy and COPD patients. However, the performance on other classes is not as good, with URTI having an F1 score of only 55%. This suggests that the model may struggle with less common conditions or those with symptoms that are difficult to distinguish from other diseases.

### Comparative analysis

In recent studies on lung sound classification, machine learning (ML) and deep learning (DL) approaches have been extensively explored. Among the ML-based methods, the highest reported accuracy reached 63.09% by Demir et al.^15^. However, DL-based approaches have demonstrated a noticeable improvement in classification accuracy over recent years. For instance, Liu et al.^2^ achieved an accuracy of 81.62% using a CNN model for a four-class classification task, although this accuracy may be considered insufficient for reliable diagnostic applications. By increasing the number of classes to six, Samiul et al.^21^ improved classification accuracy to 89.8%, likely due to their use of hybrid scalogram features and a lightweight CNN model optimized for detecting respiratory diseases. It’s possible that the scalogram-based transformation approach resulted in minor losses of detail from the raw audio, but the enhanced feature representation provided competitive performance.

In our study, we compared the proposed approach with these existing methods, specifically focusing on those using the same database and similar approaches. For a fair comparison, we have chosen only those existing works who have used the same database as ours, however, there is a discrepancy in the no. of classes considered. Many existing works do not mention the no. of classes taken and a few have considered only a limited no. of classes (2 or 4). It is worth mentioning that the model’s performance is expected to decline as the no. of classes increases. The detailed comparison study is presented in Table 7 . As can be observed in the comparison table, our approach demonstrates superior performance across various metrics, showcasing advancements in terms of model robustness and generalization. Notably, our study employs an 80/20 splitting strategy via fivefold cross-validation, dividing the dataset into training(training data was later divided into training and validation) and testing sets, allowing for a comprehensive evaluation of model performance. The TriSpectraKAN architecture employed in our model strikes a balance between model complexity and effective feature extraction, mitigating concerns related to overfitting commonly associated with excessively complex models. The utilization of both convolutional and neural network layers enables our model to capture intricate patterns within the data, leading to heightened sensitivity and specificity, with values of 97% and 92%, respectively.

**Table 7:** Summary of studies and their performance.

Study	Method	Sensitivity	Specificity	Score	Accuracy	# of Class
Serbes et al.^9^	SVM	–	–	–	49.86%	2
Jakovljević et al.^10^	HMM	–	–	–	39.56%	4
Kochetov et al.^11^	RNN	58.40%	73%	65.70%	–	4
Chambres et al.^12^	HMM SVM	–	–	42.32%	48.90%	4
Liu et al.^2^	CNN	–	–	–	81.62%	4
Ma et al.^13^	bi-ResTriSpectraKAN	58.54%	80.06%	69.30%	67.44%	4
Acharya and Basu^14^	CNN-RNN	48.63%	84.14%	66.38%	-	4
Demit et al.^15^	VGG 16 SVM	–	–	–	63.09%	4
Saraiva et al.^29^	CNN	–	40.1%	–	72.3%	4
Samiul et al.^21^	EMD-Based Scalogram	88%	88.5%	88.25%	89.8%	6
Caiwen Xu et al.^4^	Instance learning	–	–	–	43.93%	2
Subrato Bharati et al.^22^	Hybrid deep learning	63%	63%	68%	73%	7
Georgios et al.^3^	CNN-LSTM	47.37%	82.46%	64.92%	73.69%	4
Our Study	TriSpectraKAN	97%	92%	97%	93%	6

One critical aspect highlighting the generalization capabilities of our model is the training progress graph, which reveals a consistent convergence of loss and accuracy over epochs. The absence of erratic fluctuations suggests that our model is less prone to overfitting, ensuring that it generalizes well to unseen data. Additionally, the Hybrid KAN’s ability to effectively extract features from the input data contributes to the model’s overall robustness. Unlike some studies that may suffer from heavy models, our approach strikes a balance, leveraging the strengths of both convolutional and neural network layers without compromising efficiency. As a result, our model achieves a remarkable test accuracy of 93%, outperforming several existing models in the comparison.

In summary, our study not only outshines in terms of accuracy but also excels in addressing challenges related to overfitting, model complexity, and heavy architectures, presenting a more generalized and robust solution for the classification task at hand. The Hybrid KAN architecture, coupled with strategic splitting strategies, emerges as a promising avenue for achieving a well-balanced and efficient model for automated classification tasks.

### Addressing class imbalance and data scarcity

**Fig. 8:**
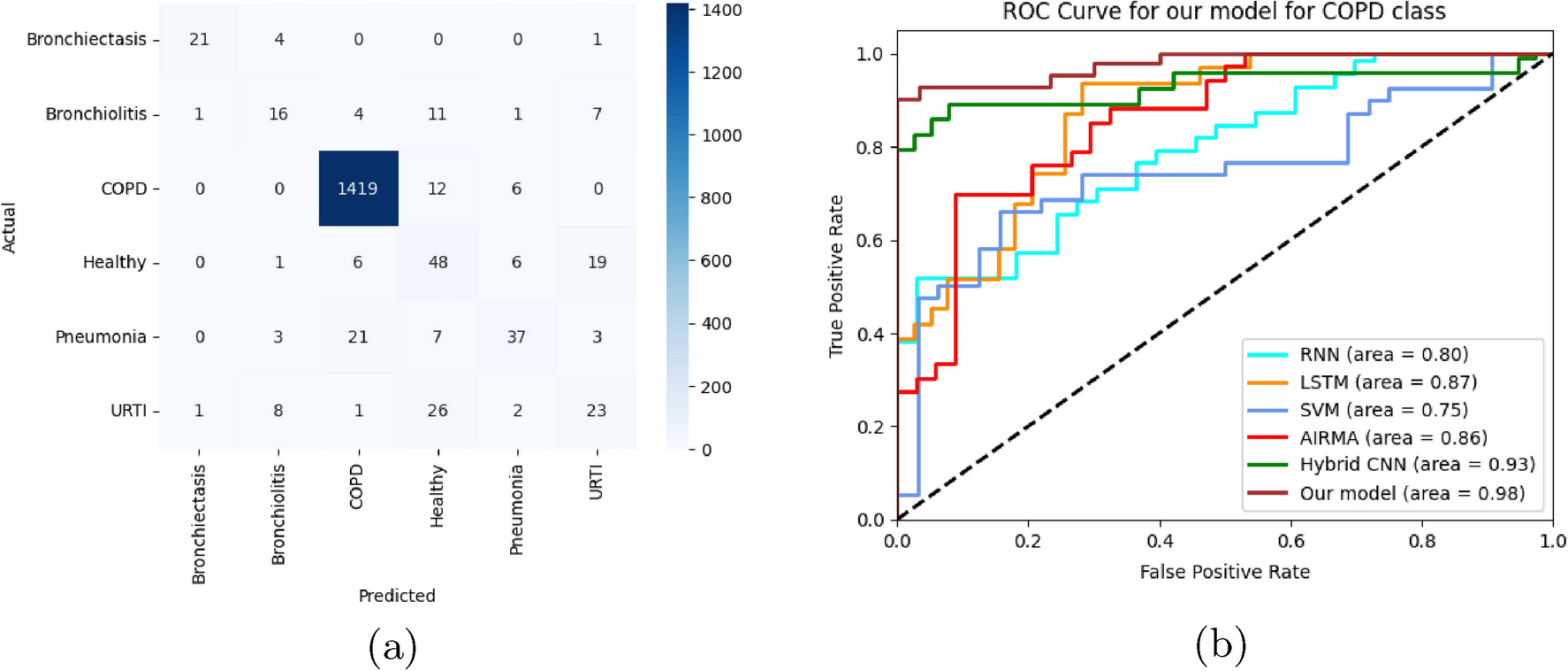
AUC-ROC plots and confusion matrix of model.

Despite the challenges presented by class imbalance and limited data samples for classes other than COPD, the provided Fig. 8a offers compelling evidence for the efficacy of our model. The confusion matrix clearly shows the data samples correctly classified and misclassified. It can be observed that COPD class has the highest correct classification rate while URTI has the lowest. This justification integrates insights from the figure’s visual elements and the F1 scores presented in the table. ROC analysis (Fig. 8) reveals the proposed model’s superior performance in COPD classification. While RNN (AUC: 0.80), LSTM (AUC: 0.87), and ARIMA (AUC: 0.86) show moderate success, SVM (AUC: 0.75) struggles with class imbalance. The Hybrid CNN achieves a strong AUC of 0.93, but our model excels with an exceptional AUC of 0.98, demonstrating near-perfect discrimination between COPD and healthy cases as well as outperforming other models.

#### Accuracy and loss curves

Flat and close training and Validation Curves: Both Fig. 6a (accuracy),b (loss) reveal curves with gentle slopes and minimal fluctuations. This indicates effective learning from the training data without succumbing to overfitting, which is crucial for generalizability. Overfitting, often characterized by sharp inclines or declines, would signify the model’s inability to adapt to unseen data.

High Validation Accuracy: The validation accuracy reaching above 0.95 in Fig. 6a suggests the model’s ability to correctly classify more than 95% of the validation set data, demonstrating its potential for good performance on unseen examples despite the imbalananced dataset.

Minimal Fluctuations: The absence of major fluctuations in the curves strengthens the model’s stability and its capacity to generate consistent predictions across different data points.

#### F1 scores

Strong COPD Performance: The outstanding F1 score of 98% for COPD indicates exceptional performance in identifying both healthy and COPD patients. This is likely due to the larger number of available COPD samples compared to other classes.

Decent Performance for Other Classes: While not as high as COPD, the F1 scores for other classes (bronchiectasis: 81%, bronchiolitis: 70%, pneumonia: 79%, URTI: 55%) suggest the model’s ability to make reasonable predictions even with fewer data points. This is particularly encouraging considering the imbalanced nature of the dataset.

It is important to recognize that the model’s performance may fluctuate among various classes owing to the data imbalance, While the results for COPD are impressive, further investigation is necessary to understand the model’s generalizability to less common conditions or those with overlapping symptoms. Additionally, exploring alternative hyperparameters and incorporating more clinical data (e.g., pulmonary function tests, demographics) could potentially enhance the model’s accuracy and robustness across all classes.

In essence, the figures and F1 scores provide strong evidence that our model achieves a commendable balance between learning from the imbalanced data and generalizing to unseen examples. This is particularly noteworthy considering the challenges posed by class imbalance and limited data samples. While further optimization and analysis are justified, the current results lay a solid foundation for the model’s potential in real-world applications.

### Results on real subjects

#### Real time analysis

To evaluate the proposed framework’s generalizability, we conducted a real-world validation using lung sound data collected from both healthy individuals and COPD patients at the pulmonary medicine department of the All India Institute of Medical Sciences (AIIMS) Raipur. Given the limited availability of COPD subjects, we recorded 15-s lung sound samples from 5 participants using a Littmann 3200 digital stethoscope. These recordings were transmitted to a laptop via Bluetooth using Littmann StethAssist software. To complement this data, we also gathered lung sound recordings from 8 healthy subjects and 2 Bronchiectasis patients in our laboratory setting.

This small-scale, real-world data collection effort aims to provide additional validation of our framework’s performance beyond standardized datasets, offering insights into its potential clinical applicability.

#### Experiment setup

The on-board implementation of the proposed hybrid CNN model leverages a Raspberry Pi 4 Model B, featuring a Broadcom BCM2711 quad-core Cortex-A72 (ARM v8) 64-bit SoC operating at 1.5 GHz with 4 GB LPDDR4 RAM. This configuration demonstrates the potential for developing a standalone point-of-care diagnostic system for COPD detection. The trained model weights were transferred to the Raspberry Pi, and essential Python libraries including Torch, Librosa, and Numpy were installed to execute the COPD classification framework.

**Fig. 9:**
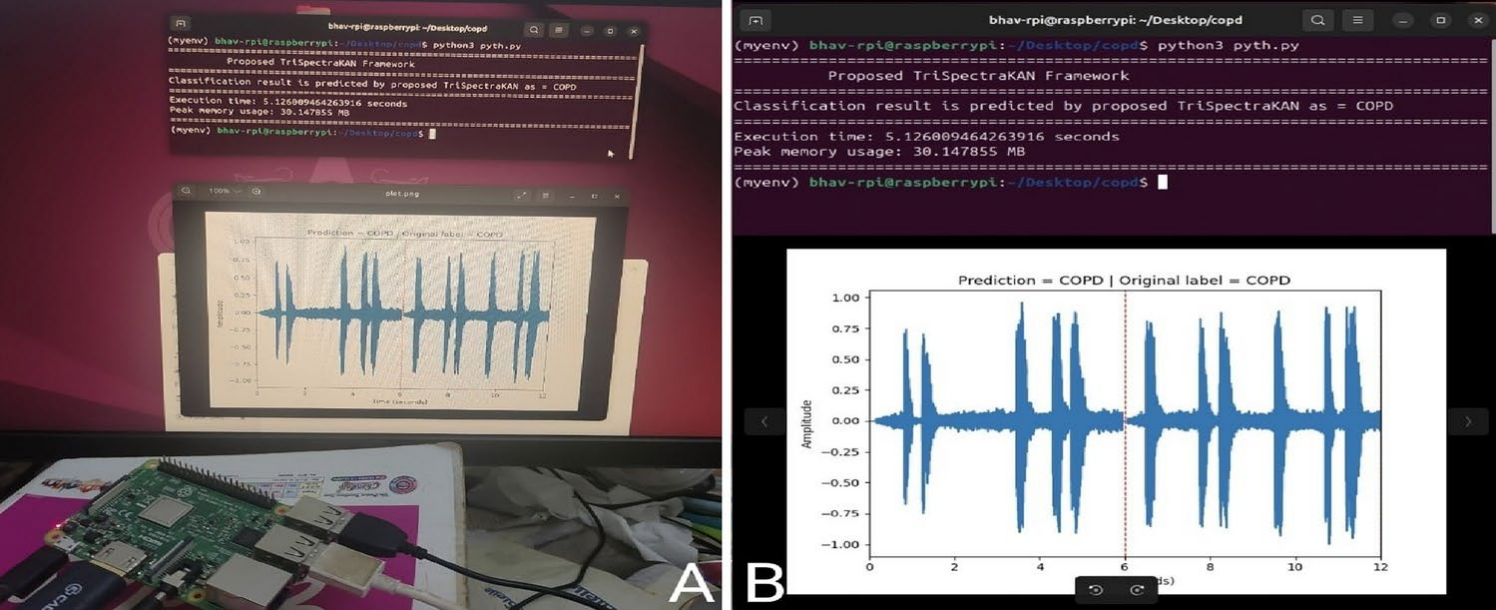
Illustrates the (**a**) on-board implementation setup and (**b**) sample classification result on Raspberry Pi terminal.

The experimental setup for on-board implementation and a sample classification result are illustrated in Fig. 9a,b, respectively. Performance analysis reveals that the proposed framework achieves a classification latency of 5.13 ± 0.23 s per lung sound segment, with a peak memory utilization of 30.20 ± 0.14 MB. These results highlight the efficiency and feasibility of deploying the model on resource-constrained edge devices, paving the way for accessible and rapid COPD diagnosis in various healthcare settings.

#### Classification performance

In this real-world validation, the deployed model accurately predicted 5 out of 5 COPD cases, 8 out of 10 healthy cases, and both Bronchiectasis cases correctly. These results demonstrate the model’s strong diagnostic capabilities across different respiratory conditions, particularly its effectiveness in identifying COPD. The slight discrepancy in healthy classifications highlights the need for further refinement in distinguishing subtle differences in lung sound patterns, but overall, the model shows promising real-world applicability.

## Conclusion

Within the scope of this investigation, we proposed a Hybrid Kolmogorov Arnold Network (KAN) model specifically designed for COPD detection, leveraging a myriad of auditory features. The results obtained from our model are promising, signaling its efficacy in precise and automated identification of Chronic Obstructive Pulmonary Disease (COPD). Our study underscores the viability of amalgamating diverse audio features within a hybrid KAN framework, demonstrating a tangible enhancement in the accuracy of COPD detection compared to traditional models.

Moreover, the successful deployment of the model on a Raspberry Pi highlights its potential as a portable, low-cost, and real-time diagnostic tool, making it particularly suitable for resource-constrained settings. The real-world validation, involving lung sound data from COPD patients, healthy individuals, and Bronchiectasis cases, further confirmed the model’s robustness, with accurate predictions across various respiratory conditions.

This study establishes a strong foundation for advancing the hybrid KAN architecture for early COPD detection. Key avenues for future exploration include optimizing hyperparameters, integrating diverse clinical data, incorporating meta-learning techniques for adaptability, validating on larger and more diverse datasets, and ultimately conducting comprehensive clinical studies to assess real-world applicability. These efforts aim to elevate the model’s accuracy and reliability, making it a valuable tool for early COPD identification, with the potential to enhance patient outcomes and reduce healthcare costs.

## Data Availability

The datasets generated and/or analysed during the current study are available in the ICBHI 2017 Challenge Database^25^, https://bhichallenge.med.auth.gr/ICBHI_2017_Challenge, Chest Wall Lung Sound Database (CWLSD) repository^19^, https://data.mendeley.com/datasets/jwyy9np4gv/3, and Respiratory Database@TR (RD@TR)^26^, https://data.mendeley.com/datasets/p9z4h98s6j/1.
